# Doxycycline to treat levodopa-induced dyskinesias in Parkinson's disease: a preliminary study

**DOI:** 10.1055/s-0043-1768668

**Published:** 2023-05-31

**Authors:** Bruno Lopes Santos-Lobato, Manuelina Mariana Capellari Macruz Brito, Ângela Vieira Pimentel, Rômulo Torres Oliveira Cavalcanti, Elaine Del-Bel, Vitor Tumas

**Affiliations:** 1 Universidade de São Paulo, Faculdade de Medicina de Ribeirão Preto, Departamento de Neurociências e Ciências do Comportamento, Ribeirão Preto SP, Brazil.; 2 Universidade Federal do Pará, Faculdade de Medicina, Laboratório de Neuropatologia Experimental, Belém PA, Brazil.; 3 Universidade de São Paulo, Faculdade de Odontologia de Ribeirão Preto, Ribeirão Preto SP, Brazil.

**Keywords:** Parkinson Disease, Dyskinesias, Doxycycline, Neuroinflammatory Diseases, Doença de Parkinson, Discinesias, Doxiciclina, Doenças Neuroinflamatórias

## Abstract

**Background** Levodopa-induced dyskinesia (LID) is a common motor complication of levodopa therapy in patients with Parkinson's disease (PD). Doxycycline is a widely used and inexpensive tetracycline with anti-inflammatory properties.

**Objective** To evaluate the efficacy and safety of doxycycline in patients with PD and LID.

**Methods** This was an open-label, uncontrolled, single-arm, single-center, phase 2 proof-of-concept study in patients with PD with functional impact of dyskinesia, which used levodopa three times daily, in a movement disorders clinic in Brazil. Participants were treated with doxycycline 200 mg/day for 12 weeks, with evaluations at baseline, week 4, and week 12 of treatment. The primary outcome measure was the change from baseline in the Unified Dyskinesia Rating Scale (UDysRS) total score at week 12, evaluated by two blinded raters. Key secondary outcomes measures were OFF time and ON time with troublesome dyskinesia in the PD home diary.

**Results** Eight patients with PD were treated and evaluated. Doxycycline 200 mg/day reduced the UDysRS total score at week 12, compared with baseline (Friedman χ ^2^ = 9.6; *p* = 0.008). Further, doxycycline reduced the ON time with troublesome dyskinesia (Friedman χ ^2^ = 10.8; *p* = 0.004) without worsening parkinsonism. There were no severe adverse events, and dyspepsia was the commonest event.

**Conclusion** In this preliminary, open-label and uncontrolled trial, doxycycline was effective in reducing LID and safe after a 12-week treatment. Further well-designed placebo-controlled clinical trials with a longer duration and a larger number of participants are needed.

**Clinical trial registration** https://ensaiosclinicos.gov.br , identifier: RBR-1047fwbf

## Resumo

**Antecedentes** A discinesia induzida por levodopa (DIL) é uma complicação motora comum da terapia com levodopa em pacientes com doença de Parkinson (DP). A doxiciclina é uma tetraciclina amplamente usada e barata, com propriedade anti-inflamatória.

**Objetivo** Avaliar a eficácia e segurança da doxiciclina em pacientes com DP e DIL.

**Métodos** Este foi um estudo aberto, não-controlado, de braço único, monocêntrico, fase 2 e de prova de conceito, em pacientes com DP e impacto funcional das discinesias, que usavam levodopa três vezes ao dia, em um ambulatório de distúrbios de movimento no Brasil. Os participantes foram tratados com doxiciclina 200 mg/dia por 12 semanas, com avaliações na base, na semana 4 e na semana 12 do tratamento. A medida de desfecho primário foi a mudança no escore total da Unified Dyskinesia Rating Scale (UDysRS) da base à semana 12, avaliada por dois avaliadores cegos. As medidas-chave de desfecho secundário fora o tempo em OFF e tempo em ON com discinesia problemática.

**Resultados** Oito pacientes com DP foram tratados e avaliados. A doxiciclina 200 mg/dia reduziu o escore total da UDysRS na semana 12, comparado com a avaliação inicial (χ ^2^ de Friedman = 9.6; *p* = 0.008). Além disso, a doxiciclina reduziu o tempo em ON com discinesia problemática (χ ^2^ de Friedman = 10.8; *p* = 0.004) sem piorar o parkinsonismo. Não houve eventos adversos graves, e dispepsia foi o evento mais comum.

**Conclusão** No presente estudo preliminar, aberto e não-controlado, a doxiciclina foi eficaz em reduzir as DIL e segura após tratamento por 12 semanas. Estudos clínicos bem-desenhados e placebo-controlados adicionais, com duração mais longa e maior número de participantes, são necessários.

## INTRODUCTION

Parkinson's disease (PD) is the second most common neurodegenerative disease, affecting ∼ 6.1 million people worldwide. 1 Levodopa is the gold standard medical therapy for motor symptoms (bradykinesia, rigidity, tremor, gait disorders). 2 However, chronic treatment with levodopa causes the onset of levodopa-induced dyskinesia (LID), hyperkinetic involuntary movements temporally associated with the use of levodopa. 3

The pathophysiology of LID is still not completely understood, but recent evidence has shown that neuroinflammation might be a leading cause of the onset of this motor complication. Animal models of dyskinesia have shown that the onset of abnormal involuntary movement in rats is associated with an increase in the density of astrocytes and activated microglia in the striatum, as well as a higher expression of proinflammatory mediators (GFAP, OX-42, TNF-α, iNOS) in neurons and glia of dyskinetic animals. 4 5 Recent CSF analyses of patients with LID in PD revealed some neuroinflammatory abnormalities: a distinct metabolic profile strongly related to the dysregulation of lipid metabolism 6 and high levels of nitrite and nitrate, which may be associated with the increased intrathecal production of nitric oxide by astrocytes and microglia. 7 These results suggest that a chronic proinflammatory state in the brain may be associated with LID onset. Although it is unclear what drives this increased inflammation, targeting neuroinflammation might be a pharmacological strategy to limit this motor complication.

Until now, the only effective medication to reduce LID in PD without worsening parkinsonism is amantadine, 8 which may cause psychosis and livedo reticularis. Many clinical trials tested the efficacy of different drugs in LID, with inconclusive results. 9 As an alternative for this shortage of treatments for LID management, repurposing drugs with proven safety may be an efficient method to bring new therapies to patients. 10 Among the most investigated drugs for repurposing, antibiotics have been tested in neurodegenerative disease due to their several mechanisms of action through modulating signaling pathways.

Doxycycline is an inexpensive second-generation semisynthetic tetracycline with easy penetration of the blood-brain barrier, reduced toxicity, longer half-lives, superior tissue fluid penetration, with exceptional bioavailability. 11 Despite being commonly used as an antibacterial drug to treat infectious diseases, doxycycline is also prescribed as an anti-inflammatory drug in the management of acne vulgaris and rosacea. Indeed, the therapeutic rationale for targeting neuroinflammation is further supported by the observation of a reduced risk of PD in individuals using tetracyclines for rosacea treatment. 12 The treatment with doxycycline is associated with few adverse events, particularly gastrointestinal symptoms and skin reactions, even after long-term administration. 13 However, there are some concerns about its long-term treatment, such as antibiotic resistance and interference on gastrointestinal microbiota.

Doxycycline has an anti-inflammatory effect in the nervous system, based on inhibition of glial activation in the substantia nigra and the striatum and suppression of metalloproteinase induction. 14 Also, doxycycline reduces the transcription of proinflammatory mediators suppressing the p38 MAPK and NF-κB signaling pathways. 11

Previously, doxycycline reduced the dopaminergic cell loss in rodent PD models 14 and inhibited microglia activation in an in vitro model of neuroinflammation. 11 Regarding LID, a recent study showed that acute and chronic intraperitoneal administration of doxycycline reduced the onset of dyskinesias in a rat model of LID. Furthermore, doxycycline reduced the expression of molecular markers of LID (FosB, COX-2, GFAP, OX-42) in the striatum of rats that developed dyskinesias. A derivative of doxycycline without antibiotic properties, COL-3, also reduced the onset of dyskinesias. 15

The exact mechanism of the doxycycline-related effect in LID is not known. Levodopa-induced dyskinesia exhibits changes in control of synaptic plasticity and neuromodulation, 16 including modifications in physical properties of synapses, synaptic protein expression, and multiple neurotransmitter systems. Doxycycline can modify neurotransmitter systems involved in the pathophysiology of LID, such as cholinergic, nitrergic, glutamatergic, and endocannabinoid systems. 17 As discussed, doxycycline may also be effective for LID in PD due to its anti-inflammatory effect on a chronic proinflammatory state in the brain. Besides lowering the expression of inflammatory markers, such as microglial and astrocytic activation, 4 5 15 doxycycline reduced the levels of TNF-α and IL-1β. 18 Therefore, doxycycline may affect the cascade of events associated with the development of LID at various sites.

Another antibiotic has shown antidyskinetic properties: ceftriaxone slowed the development of abnormal involuntary movements but did not change previously established LID, 19 acting through the expression of glutamate transporter 1. A recent clinical trial showed that intestinal decontamination with colon enemas and the luminal antibiotic rifaximin reduced LID in patients with advanced PD. 20 The authors suggested that the therapy modified gut dysbiosis, common in PD. Gut dysbiosis causes a systemic proinflammatory status that increases the brain-blood barrier permeability, promoting neuroinflammation together with bacterial products from the gut microbiota. 21 Doxycycline may also be effective in LID by reducing neuroinflammation mediated by gut dysbiosis.

Furthermore, previous animal models of PD provided evidence on the antiamyloidogenic activity of doxycycline. 22 Doxycycline inhibits the formation of toxic misfolded forms of α-synuclein oligomers through protein aggregation and blocks the seeding capacity of preformed aggregates in an in vitro and in vivo model. 23 Recently, another study in vivo confirmed these findings. 24 Previous randomized and controlled clinical trials explored the neuroprotective properties of doxycycline in Alzheimer disease 25 26 and Creutzfeldt-Jakob disease, 27 28 with conflicting results.

Considering the low cost and good safety of doxycycline and its modulating effect on neuroinflammatory mechanisms, it may represent a new therapy for LID management. Thus, we conducted an open-label, uncontrolled, single-arm, single-center, proof-of-concept phase 2 clinical trial to analyze the effects of doxycycline in LID in patients with PD.

## METHODS

### Study design and participants

We performed an open-label, uncontrolled, single-arm, single-center, phase 2 proof-of-concept study to assess the efficacy and safety of doxycycline for 12 weeks in patients with PD and LID. Participants were recruited in the Movement Disorders Unit of Ribeirão Preto Medical School, Brazil, between October 2019 and May 2021. The study was conducted following the Declaration of Helsinki and Good Clinical Practice Guidelines and was approved by the Ribeirão Preto Medical School Ethics Committee (number 3.055.052). All patients provided written informed consent.

As inclusion criteria, we selected: patients ≥18 years old; diagnosis of PD according to the UK Parkinson's Disease Society Brain Bank clinical diagnostic criteria; at least a mild functional impact of dyskinesia in the Movement Disorder Society – Unified Parkinson's Disease Rating Scale (MDS-UPDRS) 29 Part IV (item 4.2 score > 1) at screening and baseline; use of levodopa at least three times daily; antiparkinsonian medications doses unchanged for at least 4 weeks before screening and during study participation.

As exclusion criteria, we excluded: treatment of any experimental drug or intervention within 90 days before screening; moderate or severe psychotic symptoms (MDS-UPDRS Part I, item 1.2 score > 2); dementia according to MDS diagnostic criteria 30 ; severe systemic conditions (infections, malignant neoplasms, chronic kidney or liver diseases); pregnancy or lactation; history of hypersensitivity or allergic reaction to tetracyclines.

### Treatment

The study consisted of a 2-week titration phase and a 10-week maintenance phase. The dose of doxycycline 200 mg/day was chosen based on an optimized plasmatic concentration and usual dose in long-term treatments. 31 Initially, patients self-administrated one capsule of doxycycline 100 mg after breakfast once a day for 2 weeks; after 2 weeks of treatment, patients were evaluated by investigators in person; if investigators and patients reported no improvement in dyskinetic movements according to the Clinical Global Impression of Change, the dose was increased to 100 mg b.i.d at week 3. Afterwards, patients were maintained on a constant dose of doxycycline until the end of week 12, when the drug was withdrawn. All antiparkinsonian medications were maintained unchanged until the end of the study.

### Assessments and outcome measures

Patients were evaluated at baseline and weeks 4 and 12 by a movement disorders specialist. Before the baseline visit, patients were trained in filling out the PD home diary. Patients completed PD home diaries to assess their motor status every half hour for 24 hours for 3 consecutive days before baseline visit and before weeks 4 and 12 visits, and we calculated the 3-day average from home diaries. Motor status was assessed as asleep, OFF time, ON time without dyskinesia, ON time with nontroublesome dyskinesia, and ON time with troublesome dyskinesia. 32 For analyses, the sum of ON time without dyskinesia with ON time with nontroublesome dyskinesia was called “ON time without troublesome dyskinesia.” At each visit, patients were instructed not to take their regular doses of antiparkinsonian drugs 12 hours before the evaluation and to be on an empty stomach.

As the first step of the baseline visit, patients were evaluated with the MDS-UPDRS in the OFF state. Afterwards, patients ingested their regular dose of levodopa increased by 50% and were evaluated with the MDS-UPDRS Part III and the Unified Dyskinesia Rating Scale (UDysRS) 33 in the ON state when the patient started experiencing dyskinetic movements; ON state evaluations were filmed for retrospective assessment by two blinded investigators. Also, clinical and demographic data were recorded, and the completed PD home diary was reviewed. The same evaluations were performed at weeks 4 and 12, including the Clinical Global Impression of Change (CGIC) scale 34 for patients and investigators regarding the change of intensity in dyskinesias compared with baseline.

The primary outcome measure was the change from baseline in the UDysRS total score at week 12. Key secondary outcomes measures were OFF time and ON time with troublesome dyskinesia in the PD home diary. Other secondary outcomes measures included changes between baseline and week 12 visit in MDS-UPDRS Parts III and IV and total scores (ON state), ON time without troublesome dyskinesia in the PD home diary, and patient- and investigator-related CGIC.

### Statistical analysis

We performed a modified intention-to-treat analysis. Changes from baseline to weeks 4 and 12 in the outcomes (UDysRS, MDS-UPDRS, time measures from PD home diary) were assessed using the Friedman test to compare multiple repeated measures, and the Wilcoxon signed-rank test was used as a post hoc test to compare baseline and week 12. Effect sizes were calculated based on the Wilcoxon signed-rank Z value by the square root of the number of related pairs. Missing data were imputed according to the last observation carried forward method.

## RESULTS

### Study population and baseline characteristics

**Figure 1:**
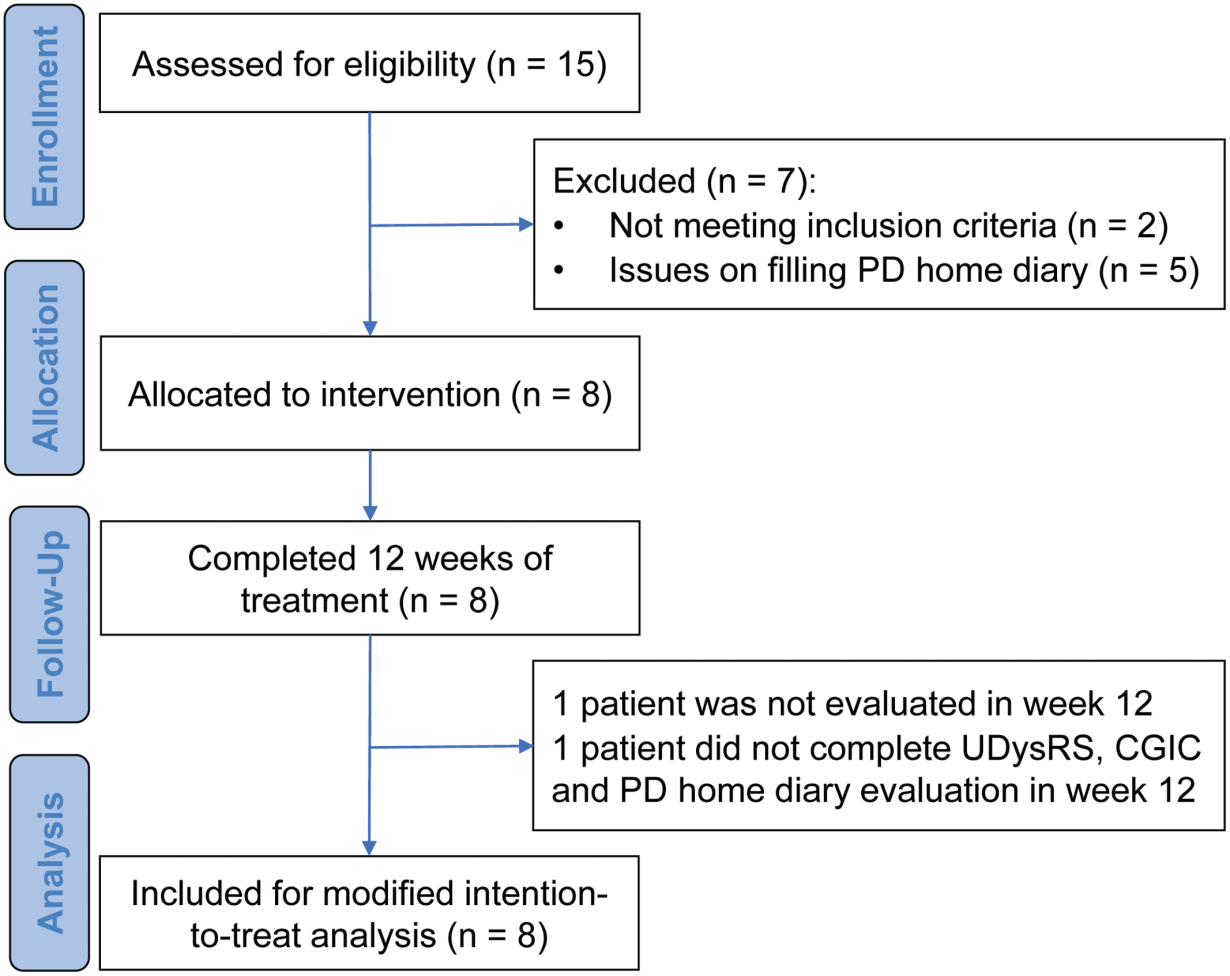
Abbreviations: CGIC, Clinical Global Impression of Change; PD, Parkinson's disease; UDysRS, Unified Dyskinesia Rating Scale. Fluxogram of the study.

A total of 15 patients were screened, and 8 patients met the inclusion criteria and were enrolled in the study ( Figure 1 ). The most common reason for failure in enrollment was that patients ( *n* = 5) had difficulties understanding the PD home diary filling procedures. Baseline demographics are provided in Table 1 . The patients were predominantly women (6 women and 2 men), had long PD and LID duration (median 17 and 10 years, respectively), and used high doses of antiparkinsonian drugs (median 1,162 mg/day). On item 4.1 of the MDS-UDPRS, 4 patients had moderate scores (item 4.1 = 3), and the other 4 had severe scores (item 4.1 = 4) in time spent with dyskinesias. On item 4.2 of the MDS-UDPRS, 2 patients had mild scores (item 4.2 = 2), 4 had moderate scores (item 4.2 = 3), and the other 2 had severe scores (item 4.2 = 4) in the functional impact of dyskinesias.

**Table 1:** Baseline clinical and epidemiological data of the eight patients

Clinical and epidemiological variable		
Male sex, % ( *n* )		25 (2)
Age at the time of evaluation (years old)		57.5 (53–65)
Age at onset of PD (years old)		41 (39–48)
Mini-Mental State Examination		27 (25–29)
Disease duration (years)		17 (8–21)
LID duration (years)		10 (5–12)
Levodopa daily dose (mg/day)		850 (724–1072)
Levodopa equivalent daily dose (mg/day)		1,162 (975–1,701)
Concomitant medication use	Use of dopaminergic agonists, % (n)	50 (4)
	Use of amantadine, % (n)	75 (6)
	Use of MAO inhibitors, % (n)	0 (0)
MDS-UPDRS	Part I	11 (6–25)
	Part II	26 (15–35)
	Part III (ON state)	32 (27–38)
	Part III (OFF state)	53 (34–61)
	Part IV	10 (8–12)
	Total score (ON state)	77.5 (60–109)
Hoehn & Yahr (ON state)		2 (2–3)
PD home diary	ON time with troublesome dyskinesia (hours)	4.17 (2–7)
	ON time without troublesome dyskinesia (hours)	7.34 (4–10)
	OFF time (hours)	4.5 (1–5)
Participants with OFF time, % ( *n* )		100 (8)
UDysRS	Historical subscore	31 (28–33)
	Objective subscore	36 (31–49)
	Total score	47 (46–59)

Abbreviations: COMT, catechol-O-methyltransferase; LID, levodopa-induced dyskinesia; MAO, monoamine oxidase; MDS-UPDRS, Movement Disorder Society-Unified Parkinson's Disease Rating Scale; PD, Parkinson's disease; UDysRS, Unified Dyskinesia Rating Scale.

Note: Values are presented as median (interquartile range) or % ( *n* ).

All patients received a daily dose of doxycycline 100 mg initially, and after 2 weeks the daily dose was increased to 200 mg due to the lack of improvement in LID. All patients completed the treatment for 12 weeks. All patients were evaluated at baseline and week 4. Two patients were not completely assessed in week 12 (1 patient did not attend the evaluation in week 12, and another patient did not complete the UDysRS, the PD home diary evaluation, and the CGIC in week 12). One patient had issues reporting the PD home diary, and these data were excluded from the analysis.

### Outcomes

**Figure 2:**
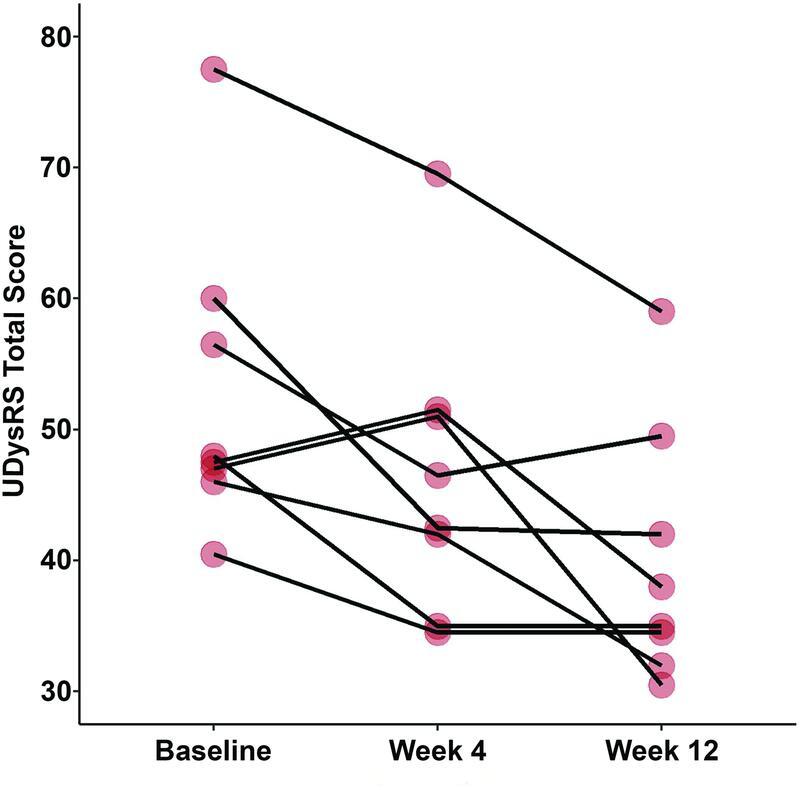
Abbreviation: UDysRS, Unified Dyskinesia Rating Scale. Change in the UDysRS total score over time (modified intention-to-treat population).

The primary efficacy analysis showed that the treatment with doxycycline was associated with a reduction in UDysRS total score until week 12 (Friedman χ ^2^ = 9.6; *p* = 0.008; week 12 compared with baseline, Wilcoxon Z = -2.521; *p* = 0.008; effect size = 0.89) ( Figure 2 , Table 2 ). There was a reduction in the historical subscore of the UDysRS until week 12 (Friedman χ ^2^ = 12.6; *p* = 0.002; week 12 compared with baseline, Wilcoxon Z = -2.524; *p* = 0.008), but not in the objective subscore (Friedman χ ^2^ = 1.31; *p* = 0.51).

**Table 2:** Efficacy results at the end of the treatment with doxycycline in the modified intention-to-treat population

General characteristics		Baseline	Week 4	Week 12	*p* -value
Primary outcome	UDysRS total score	47 (46–59)	44 (37–51)	36 (32–48)	0.008
Key secondary outcome	ON time with troublesome dyskinesia (hours)	4.1 (2–7)	1 (0.3–2)	1.33 (0.5–2)	0.004
	OFF time (hours)	4.5 (1–5)	3.5 (0.6–5)	5 (2–5)	0.36
Other secondary outcomes	MDS-UPDRS part III (ON state)	32 (27–38)	30 (24–37)	30 (24–39)	0.65
	MDS-UPDRS part IV	10 (8–12)	9.5 (8–11)	8 (7–9)	0.14
	MDS-UPDRS part IV, item 4.1	3.5 (3–4)	3 (2–3)	2.5 (2–3)	0.01
	MDS-UPDRS part IV, item 4.2	3 (2–4)	2 (2–2)	1 (1–2)	0.002
	MDS-UPDRS total score (ON state)	77 (60–109)	70 (50–88)	70 (53–88)	0.19
	ON time without troublesome dyskinesia (hours)	7.3 (4–10)	9.6 (7–13)	8.1 (8–13)	0.03

Abbreviations: MDS-UPDRS, Movement Disorder Society–Unified Parkinson's Disease Rating Scale; UDysRS, Unified Dyskinesia Rating Scale.

Note: Values are presented as median (interquartile range).

**Figure 3:**
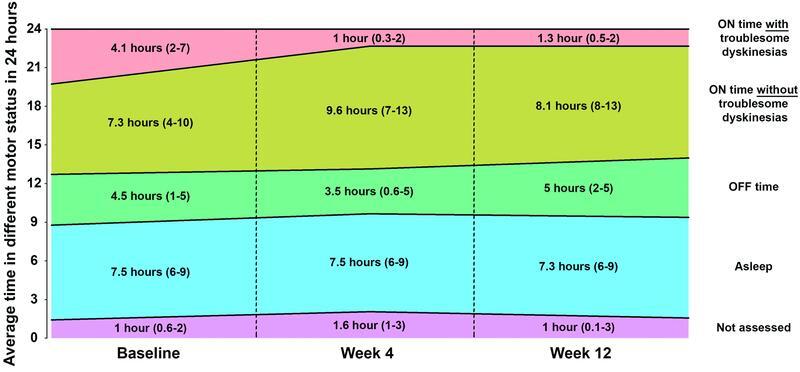
Note: Values presented as median (interquartile range). Change in the average time in different motor status according to the Parkinson's disease home diary over time (modified intention-to-treat population).

Parkinson's disease home diary measurements showed significant clinical improvements after treatment with doxycycline through 12 weeks: reduction of ON time with troublesome dyskinesias (Friedman χ ^2^ = 10.8; *p* = 0.004; week 12 compared with baseline, Wilcoxon Z = -2.36; *p* = 0.016; effect size = 0.89) and increase of ON time without troublesome dyskinesias (Friedman χ ^2^ = 6.74; *p* = 0.03; week 12 compared with baseline, Wilcoxon Z = -1.69, *p* = 0.1) ( Figure 3 , Table 2 ).

There was no worsening of parkinsonism after 12 weeks of treatment with doxycycline measured by total score and Part III of the MDS-UPDRS in the ON state ( Table 2 ). There was no modification of the MDS-UPDRS Part IV after treatment, but there were significant reductions in scores of time spent with dyskinesias (item 4.1, Friedman χ ^2^ = 8.37; *p* = 0.01; week 12 compared with baseline, Wilcoxon Z = -2.07, *p* = 0.03) and functional impact of dyskinesias (item 4.2, Friedman χ ^2^ = 13.0; *p* = 0.002; week 12 compared with baseline, Wilcoxon Z = -2.56; *p* = 0.01).

The CGIC scale results showed an overall agreement between impressions from investigators and patients, with all patients assessed as improved by investigators after treatment with doxycycline at week 12, and 7 of 8 patients self-reported themselves as improved (87.5%).

### Safety

Overall, 6 patients (75%) reported at least 1 adverse event, 1 (12.5%) patient had a moderate adverse event (abdominal pain throughout treatment duration in a patient with previous gastritis), and no patient had an adverse event that led to discontinuation of the therapy or serious adverse events. Most adverse events were grade 1 or 2 according to the Common Terminology Criteria for Adverse Event (version 5.0). Most of these adverse events were drug-related, and dyspepsia ( *n* = 4; all grade 1) and abdominal pain ( *n* = 2; one grade 1 and one grade 2) were the most common events. Nausea ( *n* = 1), headache ( *n* = 1), and somnolence ( *n* = 1) were also described.

## DISCUSSION

In the present preliminary, open-label and uncontrolled trial, doxycycline 200 mg daily for 12 weeks reduced LID frequency, severity, and functional impact in patients with PD using levodopa, as assessed by UDysRS, PD home diary, MDS-UPDRS, and CGIC. Also, doxycycline increased ON time without troublesome dyskinesias, without worsening motor and nonmotor symptoms. From baseline, the UDysRS total score was 11 points lower and ON time with troublesome dyskinesias was 2.77 hours shorter at week 12. At baseline, all patients with PD had LID during at least 50% of their waking day (MDS-UDPRS item 4.1 scores 3 and 4), and 75% of patients had LID, which impeded their activities (MDS-UDPRS item 4.2 scores 3 and 4); after doxycycline (week 12), only 50% of patients had LID during at least 50% of waking day, and no patient had LID which impeded activities.

The treatment with doxycycline 200 mg daily showed no serious adverse events. Dyspepsia, the most frequently observed adverse event, was mostly mild and did not cause discontinuation of the drug. Only one patient had moderate adverse effects (abdominal pain persisting for 3 months). A previous study with doxycycline 200 mg/day for 12 weeks reported photosensitivity and nausea as the main adverse effects, but without abdominal pain. 35

UDysRS total score has been suggested as the preferred primary outcome for clinical trials on LID. 36 Minimal clinically important difference (MCID) in LID has been explored recently. For UDysRS, there are established MCIDs for the historic subscores (Part 1 - ON dyskinesia, - 2.1 points; Part 2 - OFF dyskinesia, - 1.8 points) 37 and the impairment subscore of the scale (Part 3, - 2.32 points). 38 Our study showed a clinically important reduction of 10.5 points for the historic subscore Part 1 at week 12, but without significant reductions in Parts 2 and 3 of the UDysRS.

Our results can be put into perspective with other studies. However, we must be cautious about comparing our open-label, uncontrolled, single-arm, phase 2 study with well-powered double-blinded, randomized, and controlled phase 3 clinical trials. The 274 mg extended-release oral formulation of amantadine (ADS-5102) once daily at bedtime was approved by the FDA in 2017 for the treatment of LID after positive results from two phase 3 randomized and controlled trials (EASE LID 39 and EASE LID 3 40 ). These studies showed a reduction of - 15.9 39 and - 20.7 points 40 in the UDysRS total score after ADS-5102 at week 12, as well as our doxycycline trial indicated a reduction of - 11 points in the UDysRS at week 12. Also, ON time without troublesome dyskinesias increased in 3.6 39 and 4 hours 40 after ADS-5102 at week 12, and doxycycline increased the ON time without troublesome dyskinesias by 2.77 hours. A recent pooled analysis of these two studies calculated the magnitude of reduction of ON time with dyskinesias after ADS-5102 as a Cohen d effect size of 0.49. 41 For comparison, our effect size for reduction of ON time with troublesome dyskinesias after doxycycline was 0.83.

As limitations, our study was not randomized and controlled, and the small size may have underpowered the results. The increase of 50% of the scheduled levodopa dose may mask the antidyskinetic effect of doxycycline in the objective subscore of the UDysRS. Also, the short duration of treatment (12 weeks) may not be adequate to measure efficacy and safety for oral doxycycline 200 mg daily in LID. An additional group for doxycycline in a smaller daily dose could be included in further studies, considering that its antidyskinetic effect may not be associated with an antibiotic dose. The reduction of LID in rats after treatment with subantibiotic doses of doxycycline and with its derivative without antibiotic effect COL-3 might be used to reduce the risk of adverse events related to long-term therapy. 23

Also, the absence of a control group in our study might cause an improvement in LID due to the placebo effect. According to the UDysRS, the placebo caused a reduction of 7 to 8 points in the total score on EASE LID 39 and EASE LID 3 40 studies after 12 weeks. The PD home diary showed that placebo-treated patients had a reduction of 2.1 hours of ON time with troublesome dyskinesias. 41 The duration and severity of dyskinetic movements may improve by 25% in the placebo group. 42 Thus, we cannot exclude that the potential antidyskinetic effect of doxycycline may be merged with a relevant placebo impact in our results.

In conclusion, the present preliminary, open-label and uncontrolled clinical trial first revealed that doxycycline could directly modulate LID in accordance with previous preclinical studies. Our findings warrant further investigation for treating LID in PD with doxycycline and other tetracyclines. Therefore, new well-designed placebo-controlled clinical trials with a longer duration and a larger number of participants would be the next logical step in this field.
